# Dosimetric impact of intra-fraction prostate motion under a tumour-tracking system in hypofractionated robotic radiosurgery

**DOI:** 10.1371/journal.pone.0195296

**Published:** 2018-04-05

**Authors:** Yuhei Koike, Iori Sumida, Hirokazu Mizuno, Hiroya Shiomi, Keita Kurosu, Seiichi Ota, Yasuo Yoshioka, Osamu Suzuki, Keisuke Tamari, Kazuhiko Ogawa

**Affiliations:** 1 Department of Radiation Oncology, Osaka University Graduate School of Medicine, Suita, Osaka, Japan; 2 Miyakojima IGRT Clinic, Miyakojima-ku, Osaka, Japan; 3 Department of Radiology, Osaka University Hospital, Suita, Osaka, Japan; 4 Department of Carbon Ion Radiotherapy, Osaka University Graduate School of Medicine, Suita, Osaka, Japan; North Shore Long Island Jewish Health System, UNITED STATES

## Abstract

For CyberKnife-mediated prostate cancer treatment, a tumour-tracking approach is applied to correct the target location by acquiring X-ray images of implanted fiducial markers intermittently. This study investigated the dosimetric impact of intra-fraction prostate motion during CyberKnife treatment. We retrospectively analyzed 16 patients treated using the CyberKnife (35 Gy delivered in five fractions). Using log files of recorded prostate motion, the intra-fraction prostate motion was simulated. We defined the worst-case intra-fraction prostate motion as the difference between pre- and post-deviation on log files and shifted structure sets according to the corresponding offsets for each beam. The dose–volume indices were calculated and compared with the original plan in terms of clinical target volume (CTV), planning target volume (CTV plus a 2-mm margin), rectum, bladder, and urethra. Prostate motions of >3, >5, and >10 mm were observed for 31.3, 9.1, and 0.5% of the 1929 timestamps, respectively. Relative differences between the simulated and original plans were mostly less than 1%. Although significant decreases were observed in D_50%_ and D_98%_ of the target, absolute dose differences were <0.1 Gy compared with the planned dose. The dosimetric impact of intra-fraction prostate motion may be small even with longer treatment durations, indicating that the tumour tracking using the CyberKnife could be a robust system for examining prostate motion.

## Introduction

Hypofractionated stereotactic body radiation therapy (SBRT) is an attractive strategy for prostate cancer in terms of radiobiology in that the alpha-beta ratio for prostate cancer is small (1.5 Gy) [1,2]. Because of the high fractional dose and small fractions compared with the conventional fractionated dose (2 Gy per fraction), a slight positional error may result in urinary and rectal side effects as well as insufficient dose coverage [3]. Therefore, irradiation accuracy is more important for hypofractionated SBRT than for fractionated regimens such as intensity-modulated radiation therapy (IMRT). Inter- and intra-fraction prostate motion may result in inaccurate beam delivery, and intra-fraction motion may be larger because of the longer duration of treatment and smaller fractions of radiation [4]. To minimize the uncertainty of prostate motion, various image-guided techniques have been developed and introduced for hypofractionated SBRT, such as cone-beam computed tomography (CT), electronic portal imaging device, ultrasound, and electromagnetic transponders, which are classified as real-time or nearly real-time monitoring systems [5–9].

Hypofractionated SBRT using CyberKnife (Accuray Inc., Sunnyvale, CA) allows a high conformal dose distribution to moving targets with sub-millimetre accuracy by monitoring prostate motion during beam delivery [10]. To compensate for intra-fraction prostate motion, the CyberKnife employs a tumour-tracking system using orthogonal kilovoltage X-ray images to detect fiducial markers, which act as surrogates of prostate motion. Prostate motion from the planned position is calculated during the acquisition of each image and corrected automatically, and the CyberKnife continues to irradiate the corrected position until the next image acquisition. Although the tumour tracking via orthogonal X-ray imaging is essential to reduce planning target volume (PTV) margins with respect to the intra-fraction motion, this nearly real-time monitoring might miss prostate motion that occurs between two consecutive image acquisitions.

Prostate motion is known to be caused by the influence of surrounding structures such as rectum and bladder, and it is correlated with the status of rectal filling by gas and stool [11–14]. If surrounding organs suddenly move immediately after correction, the CyberKnife system cannot detect prostate motion during the next image acquisition, potentially resulting in insufficient dose coverage and unexpectedly high doses to organs at risk (OARs). Rectum and bladder motion vary from day to day and even during beam delivery [15]. The degree of prostate motion differs among individuals and large intra-fraction prostate motion (>5 or >10 mm) can occur during the delivery of each fraction [15–17]. In addition, intra-fraction prostate motion increases with time [9,12,18]. Based on these facts, knowing and understanding the impact of prostate motion between consecutive image acquisitions are valuable under the nearly real-time correction of the CyberKnife.

In this study, we introduced actual prostate motion data to the dose calculation and investigated the dosimetric impact of intra-fraction prostate motion for hypofractionated CyberKnife treatment via the dose–volume comparison.

## Materials and methods

### CyberKnife treatment protocol for prostate cancer

Under an exemption status from our institutional review board, CyberKnife G4 linear accelerator has been employed in a phase II/III clinical trial for hypofractionated radiotherapy for patients with low- and intermediate-risk prostate cancer. This study was approved by the Institutional Review Board of Osaka University Hospital, and written informed consent was obtained from all patients. We retrospectively analyzed 16 patients treated in this clinical trial between December 2014 and May 2015.

For each patient, three fiducial markers were implanted into the prostate before a planning CT (pCT). In our institution, pCT was performed with a urinary catheter balloon that was inserted immediately before pCT because of its invisibility, which allowed us to delineate and evaluate the urethral dose accurately. After urinating via a catheter, the bladder was filled with 100 ml of saline to ensure the reproducibility of bladder shape before pCT and individual treatment fractions. The urinary catheter balloon was extracted after pCT and inserted again immediately before the first treatment session. Clinical target volume (CTV) comprised the prostate and part of the seminal vesicle (SV) plus a 3-mm margin, excluding a 1-mm margin on the rectum side, and the PTV was defined as the CTV plus a 2-mm margin. Additionally, a 2-mm margin was added around the urethra for the planning organ at risk volume (PRV). All treatment plans were calculated using a MultiPlan (version 4.6.1; Accuray Inc.) treatment planning system (TPS). The prescription dose was 35 Gy delivered in five fractions to cover at least 95% of the PTV. Dose constraints of the surrounding organs are summarized in Table 1. For all patients, the pre-treatment CT was acquired before every fraction. This was compared against the pCT to check whether the relative position relationships of the fiducial markers with the prostate and urethra were stable.

**Table 1:** Dose constraints for the CyberKnife prostate plan in this trial.

Structure	Constraint
Rectum	D_2cc_ < 35 Gy
	D_5cc_ < 30 Gy
	V_50%_ < 40%
Bladder	D_10cc_ < 35 Gy
	V_50%_ < 35 cc
	V_100%_ < 5 cc
Urethra	D_10%_ < 50 Gy
	D_30%_ < 45 Gy
	D_Max_ < 107%
Femoral head	V_40%_ < 5%

Although the CyberKnife is capable of performing six-dimensional (6D) correction, we only used three-dimensional (3D) correction in consideration of the sufficient accuracy of 3D correction and the collision risk of 6D correction between the linac head and the wall or floor of the treatment room [19,20].

### Fiducial tracking and intra-fraction motion data

Intra-fraction prostate motion was recorded in log files saved on the CyberKnife system. The log files were generated after each fraction, and contained treatment information for each beam, including the monitor unites, beam and node number, and the displacement of target position from pCT measured by a pair of orthogonal X-ray images at every timestamp. During the delivery of each fraction, the target position expressed by the centre of the mass of three fiducial markers in the anterior-posterior (AP), right-left (RL), and superior-inferior (SI) directions was measured as designated by the timestamps, and deviation from a reference position in the digitally reconstructed radiograph at pCT was kept in a log file. In this study, intra-fraction prostate motion was defined as the displacement of the centre of the mass of three fiducial markers between the timestamps and the pCT. For all 16 patients, the interval of image acquisition was adjusted by an operator based on the status of the patient’s motion. The mean fraction time (standard deviation, SD) was 38.2 (16.1) min and the total number of orthogonal kV pairs in five fractions was 121 (range, 101–141) on average for each patient. The X-ray frequency was approximately 70 s (range, 10–478 s) in this study on average [21].

The log files were exported from the CyberKnife system for the analysis of intra-fraction prostate motion. The mean, SD, maximum, minimum value, root mean square errors for the AP, RL, and SI directions and 3D vector length were calculated.

### Intra-fraction prostate motion simulation

Prostate motion data in log files were incorporated into the dose calculation to evaluate the impact of intra-fraction prostate motion using ShioRIS2.0 (RAD Lab Co., Ltd., Osaka, Japan), which can implement the dose calculation using DICOM RT files exported from MultiPlan [22–24]. ShioRIS employs a ray tracing algorithm, which is also used in CyberKnife TPS. The dose difference between ShioRIS and CyberKnife TPS was within 2% for the maximum, mean, and minimum dose to the target [22]. ShioRIS2.0 can simulate intra-fraction motion by shifting DICOM RT structures using offsets for each beam and accumulating dose value at different grid points corresponded to the offsets. We simulated the worst-case scenario in which the position of the prostate changed after image acquisition.

**Fig 1:**
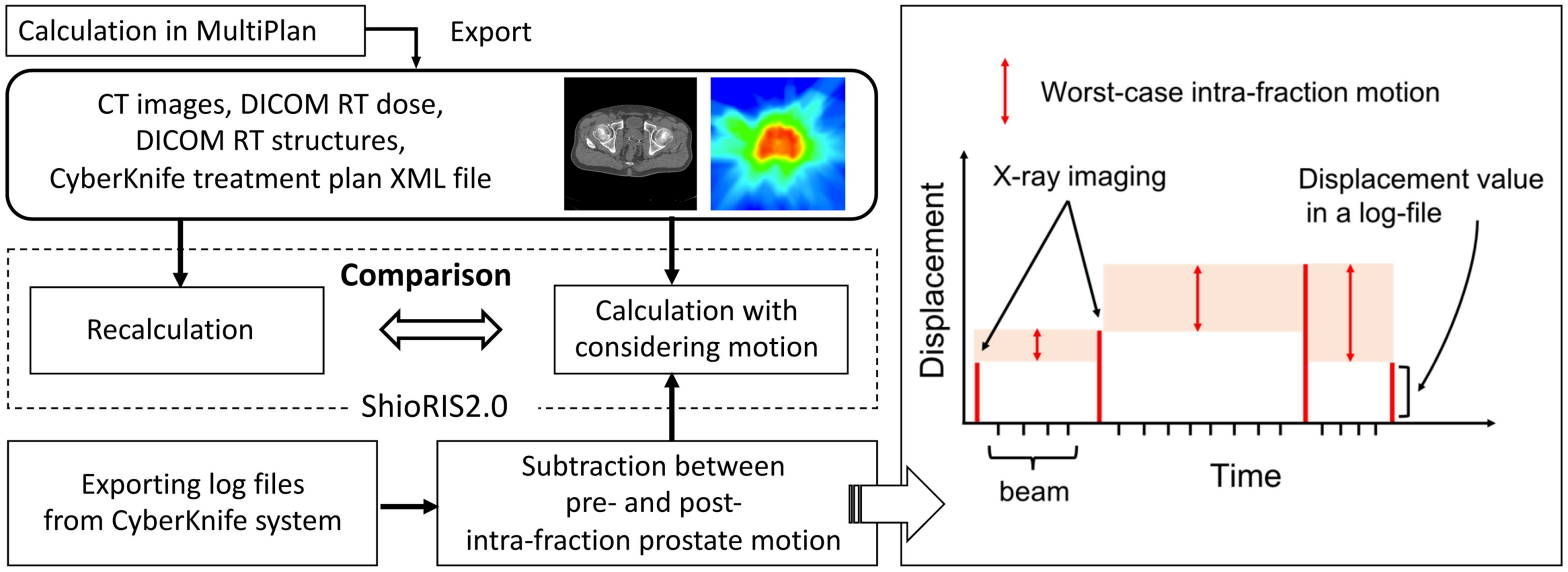
A schema of intra-fraction prostate motion simulation using log files. A flowchart of intra-fraction prostate motion simulation using ShioRIS2.0 (left) and a diagram showing the worst-case intra-fraction motion during tracking irradiation based on log file analysis (right). The worst-case motion scenario that prostate had moved to the next detected position immediately after X-ray image acquisition was simulated by subtracting intra-fraction prostate motion between pre- and post-deviation on log files.

Fig 1 presents a flowchart of this simulation. First, all treatment plans for 16 patients were calculated using MultiPlan. We exported CT images, DICOM RT dose, DICOM RT structures, and the CyberKnife treatment plan XML file from MultiPlan and imported them into ShioRIS2.0. Then, treatment plans were re-calculated using ShioRIS2.0 as reference plans with no motion. Second, we exported log files from the CyberKnife system. To simulate the worst-case intra-fraction prostate motion between two consecutive acquisitions, we calculated the difference between pre- and post-deviations on log files for each fraction and each patient and defined this differential value as the worst-case intra-fraction prostate motion between two timestamps during tracking irradiation. These motion data were imported into ShioRIS2.0, and treatment plans were calculated in consideration of intra-fraction motion. This motion plan was compared with the reference plan calculated with ShioRIS2.0. The differences between dose–volume indices with and without the motion were compared with respect to D_2%_ (near-maximum dose), D_50%_, D_98%_ (near-minimum dose), and homogeneity index (HI) for CTV and PTV, D_5cc_ and V_50%_ for rectum, D_10cc_ and V_100%_ for bladder, and D_10%_ and D_30%_ for urethra. In accordance with ICRU report 83 [25], HI was defined as follows:

$$HI=\frac{D_{2\%}-D_{98\%}}{D_{50\%}},$$

where D_*x*_% was the dose that covered *x*% of the target volume.

### Statistical analysis

All statistical analyses were performed using JMP Software (SAS Institute Inc., Cary, NC). The normality of the data was tested using the Shapiro–Wilk test. Depending on the normality, the differences between dose–volume indices with and without considering motion were compared using a paired *t*-test or the Wilcoxon signed-rank test. Statistical significance was set at a 5% level.

## Results

### Characteristics of intra-fraction prostate motion

**Fig 2:**
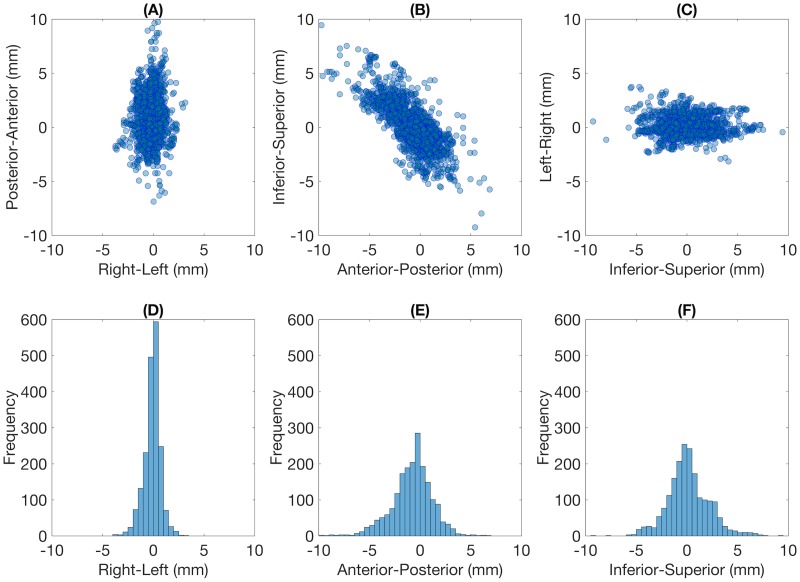
Characteristics of intra-fraction prostate motion for 1929 timestamps. Two-dimensional scatter plots in the (A) axial, (B) sagittal, and (C) coronal planes, and histograms in the (D) right-left, (E) anterior-posterior, and (F) inferior-superior directions. The width of bins was 0.5 mm.

For all 1929 timestamps, two-dimensional scatter plots showing intra-fraction prostate motion in the axial, sagittal, and coronal plane and histograms in the RL, AP, and SI directions are shown in Fig 2. The statistical characteristics of prostate motion and the worst-case intra-fraction motion during tracking irradiation based on log file subtraction are summarized in Table 2. The mean absolute shifts per timestamp were 1.54 ± 1.37, 0.59 ± 0.56, and 1.59 ± 1.44 mm in the SI, RL, and AP directions, respectively. The mean 3D vector length was 2.57 ± 1.77 mm. The 3D vector length exceeded 3, 5, 7, and 10 mm in 31.3, 9.1, 2.7, and 0.5% of the cases.

**Table 2:** Summary of prostate motion per timestamp on log files, the worst-case motion during tracking irradiation simulated in this study in the SI, RL, and AP directions and the 3D vector length.

	Intra-fraction motion (mm)				Worst-case motion (mm)			
	SI	RL	AP	3D vector length	SI	RL	AP	3D vector length
Mean	−0.15	−0.09	0.79	2.53	0.03	−0.01	−0.02	0.88
SD	2.06	0.81	1.99	1.77	0.91	0.35	1.04	1.25
Max	9.25	3.15	9.73	13.56	5.81	2.83	10.13	13.73
Min	−9.43	−3.73	−6.87	0.10	−9.48	−3.09	−8.68	0.00
RMSE	2.06	0.82	2.15	-	0.91	0.35	1.04	-

The sign of intra-fraction motion in the SI, RL, and AP directions is consistent with the coordinates in Fig 2.

SI, superior-inferior; RL, right-left; AP, anterior-superior; 3D, three-dimensional; SD, standard deviation; Max, maximum; Min, minimum; RMSE, root mean square error.

### Dosimetric impact of intra-fraction motion

Table 3 summarizes the comparison of dose–volume indices between with and without consideration of intra-fraction prostate motion. Relative differences were less than 1% excluding V_100%_ of the bladder and D_30%_ of the urethra; however, the differences were not significant. Statistically significant decreases were found for D_50%_ of the CTV and D_50%_ and D_98%_ of the PTV compared with the original plan.

**Table 3:** Comparison of dose–volume indices between with and without consideration of intra-fraction prostate motion.

		Without motion (Mean ± SD)	With motion (Mean ± SD)	Difference between mean values	Relative error (%)	p-value
CTV	D_2%_ (Gy)	42.78 ± 1.87	42.72 ± 1.82	−0.06	−0.14	0.12*
	D_50%_ (Gy)	39.15 ± 1.10	39.07 ± 1.06	−0.08	−0.20	0.011
	D_98%_ (Gy)	35.24 ± 0.58	35.16 ± 0.61	−0.08	−0.22	0.051
	HI	0.19 ± 0.05	0.19 ± 0.05	0.00	0.42	0.50*
PTV	D_2%_ (Gy)	42.59 ± 1.85	42.53 ± 1.79	−0.07	−0.15	0.083*
	D_50%_ (Gy)	38.64 ± 0.99	38.55 ± 0.96	−0.09	−0.22	0.007
	D_98%_ (Gy)	33.73 ± 0.65	33.63 ± 0.72	−0.10	−0.29	0.005*
	HI	0.23 ± 0.05	0.23 ± 0.06	0.00	0.49	0.13*
Rectum	D_5cc_ (Gy)	26.95 ± 2.45	26.86 ± 2.35	−0.10	−0.32	0.25*
	V_50%_ (%)	30.47 ± 3.69	30.45 ± 3.74	−0.02	−0.08	0.71
Bladder	D_10cc_ (Gy)	27.32 ± 2.68	27.26 ± 2.67	−0.06	−0.20	0.25*
	V_100%_ (cc)	1.88 ± 1.15	1.81 ± 1.12	−0.07	−6.00	0.11
Urethra	D_10%_ (Gy)	37.29 ± 1.75	37.24 ± 1.74	−0.05	−0.14	0.31
	D_30%_ (Gy)	28.86 ± 7.49	28.95 ± 7.14	0.09	1.18	1.0*

*p-value from non-parametric test.

CTV, clinical target volume; PTV, planning target volume; SD, standard deviation; HI, homogeneity index.

## Discussion

Intra-fraction prostate motion was previously investigated using various detection techniques, including electromagnetic tracking devices, ultrasound, magnetic resonance imaging (MRI), and two orthogonal kilovoltage X-ray sources.

Li et al. [8] used electromagnetic transponders to assess prostate motion for 35 patients who underwent IMRT. They reported that the mean (±1 SD) prostate motion in the SI, RL, and AP directions were 0.3 ± 0.7, 0.0 ± 0.3, and 0.4 ± 0.6 mm, respectively. Similarly, Langen et al. [9] used electromagnetic transponders for IMRT to localize prostate position in 17 patients and reported motions of >3 and >5 mm for 13.6 and 3.3% of the total treatment duration. Baker et al. [26] studied prostate motion in 10 patients treated via volumetric modulated arc therapy using a transperineal ultrasound system for prostate tracking. They reported that the mean 3D vector length of prostate motion during beam delivery for approximately 2–2.5 min was 0.9 ± 0.6 mm. Mah et al. [17] used cine MRI for 42 patients to determine intra-fraction prostate motion and demonstrated that the prostate motion (mean ± SD) during an approximately 9-min scan were 0.0 ± 3.4, 0.0 ± 1.5, and 0.2 ± 2.9 mm in the SI, RL, and AP directions. The previously mentioned prostate motion was observed during a relativity shorter duration compared with that for CyberKnife SBRT. In CyberKnife treatment, Xie et al. [16] investigated intra-fraction prostate motion for 21 patients using log files and reported that the mean displacements (absolute) were 1.55 ± 1.28, 0.87 ± 1.17, and 1.80 ± 1.44 mm in the SI, RL, and AP directions and the 3D vector length was 2.61 ± 1.94 mm.

We also analyzed log files using the nearly real-time monitoring system. The mean prostate deviation and SD coincided with the findings of published studies [8,16,17]. However, with respect to the 3D vector length, large prostate motion was observed more frequently than that found with shorter durations. In general, hypofractionated radiotherapy prolongs the treatment time because of the large dose delivered in each fraction. Moreover, the CyberKnife requires additional time to deliver irradiation from multiple nodes, to move the robotic arms, and to acquire the X-ray image for correction. Because prostate motion time-dependently increases, the observed prostate motion (Table 2) might be a reasonable result even though large prostate motion was frequently detected because of the long duration of CyberKnife treatment (approximately 40 min in this study), which is approximately 2–3-fold longer than that of conventional radiotherapy or IMRT [9,12,18]. In addition, Smeenk et al. [27] reported that the rate of prostate motion >3 mm using the Calypso system (Varian Medical Systems, Palo Alto, CA) was 1.4% for the first 2.5 min of treatment, compared to 18% for a 10-min duration, which is assumed to support our findings.

We investigated whether for long treatment time periods, during which motion is not monitored between X-ray image acquisitions on the CyberKnife tracking system, a large prostate motion was acceptable. We simulated the actual delivered dose in consideration of intra-fraction prostate motion by adapting the patient’s own motion data in log files to the patient retrospectively. As shown in Table 3, the impact of prostate motion was presumed to be small and negligible. The relative error of most dose–volume indices was less than 1% even in the worst-case scenario. Although significant differences were observed in CTV and PTV, the absolute dose difference was less than 0.1 Gy. Regarding OARs, a large decrease was observed for V_100%_ of the bladder, which might be attributed to the small value of V_100%_. Regarding the urethra, the dose constraints were adequately satisfied even when intra-fraction prostate motion was considered. This might be due to the visualization of urethra using a catheter, which allowed us to add the PRV margin around the urethra. Generally, the dosimetric impact of intra-fraction prostate motion is assumed to be small in conventional fractionated radiotherapy because of its nature, including randomness throughout the fractionated regimen. However, it may cause clinically important errors in hypofractionated treatment, as mentioned by Hoogeman et al. [28]. Although CyberKnife is an extreme hypofractionated treatment in which 35–40 Gy of radiation is delivered in five fractions, the impact of prostate motion between X-ray acquisitions was presumed to be negligible (Table 3). This may be attributable to the unique characteristics of prostate motion and the CyberKnife. The CyberKnife provides a conformal dose distribution using numeric beams with small monitor units from various nodes. Additionally, prostate randomly moves around the reference position during pCT. Therefore, we considered that the random motion of the prostate resulted in “blurring” coupled with the non-coplanar irradiation of the CyberKnife. Consequently, the dose at the edge of the target might be blurred, resulting in decreases of D_50%_ and D_98%_ for the target.

The findings from this study suggest that the dosimetric impact of intra-fraction prostate motion may be small with the mean timestamp interval about 70 s. As Xie et al. [16] demonstrated that the rate of large motion decreased as the timestamp interval was less, the shorter interval can improve the beam deliverability. However, intra-fraction prostate motion may be independent of the timestamp interval because of the random and sporadic characteristic of prostate motion. Considering the extension of treatment time and an increase of radiation exposure to normal tissue in addition to our findings, too short interval may not be necessary for CyberKnife prostate treatment.

The limitations of this method include the fact that rotational correction was not applied and prostate deformation was not considered. van de Water et al. [4] investigated the effect of CyberKnife correction strategies for prostate cancer. They demonstrated that when using both translational and rotational correction under a 3-mm PTV margin, more than 99% of the treatment achieved V_100%_ >98% for CTV with 60–180 s X-ray acquisition intervals. Using translational correction only, treatments in which V_100%_ exceeded 95% were able to achieve 98% CTV coverage. Concerning the deformation, prostate motion can be affected by rectal motion, and the variation of rectal motion differs depending on its site [11,29]. Thus, our method is limited in that we dealt with the prostate as a rigid body by shifting the whole structure. Although prostate rotation and deformation could reflect the secondary correction and they are much smaller than translation [30,31], further research of the impact of rotation and deformation on hypofractionated CyberKnife SBRT using deformable image registration techniques and fully 6D correction should be conducted.

Another limitation was that the intra-fraction SV motion was not considered. For the intermediate- and high-risk patients, the target volume usually includes the SV. Previous studies investigated the intra-fraction SV motion using cine MRI [32] and cone beam CT [33]. They demonstrated that the intra-fraction SV motion in the SI direction is larger than the prostate motion, and the prostate and SV did not move in unison. Although CTV included SVs in this study, we added an isotropic margin around CTV and shifted the prostate and SV by the same displacement as the intra-fraction prostate motion in the simulation. Because the SV is invisible in the orthogonal kV images, an additional margin to account for intra-fraction SV motion should be considered from the clinical standpoint.

## Conclusion

This study investigated the intra-fraction prostate motion under the nearly real-time correction of CyberKnife by incorporating motion data into the dose calculation. The dosimetric impact of intra-fraction prostate motion may be small even with the longer treatment duration, indicating that the tumour tracking of the CyberKnife could be a robust system for assessing prostate motion. We concluded that intra-fraction prostate motion may be acceptable for the CyberKnife, as it features a conventional fractionated schedule.
